# Peritoneal Metastases

**DOI:** 10.1155/2012/695351

**Published:** 2012-09-12

**Authors:** Yutaka Yonemura, Yan Li, Paul H. Sugarbaker, Pompiliu Piso

**Affiliations:** ^1^Haruki-Moto-Machi, Kishiwada City, Osaka 596-0032, Japan; ^2^Department of Oncology, Zhongnan Hospital of Wuhan University, No. 169, Donghu Road, Wuchang District Wuhan 430071, China; ^3^Program in Surface Malignancy, Washington Hospital Center, 106 Irving St, NW, Washington, DC 20010, USA; ^4^Chefarzt Klinik für Allgemein-und Viszeralchirurgie Krankenhaus Barmherzige Brüder, Regensburg Prüfeninger Straße 86, 93049 Regensburg, Germany

In the past, peritoneal metastases (PM) were considered as a final stage of cancer, and patients were offered a palliative chemotherapy or at best supportive care. In the early 1990s, some surgeons developed a new therapeutic alternative based on the combined treatment. In this curative intent, the macroscopic disease was treated with cytoreductive surgery (CRS) followed by treating residual microscopic disease with a direct intraabdominal application of intraperitoneal chemotherapy using peroperative hyperthermic intraperitoneal chemotherapy (HIPEC) and/or under normothermia of early postoperative intraperitoneal chemotherapy (EPIC). In 2003, prolonged survival of patients affected by peritoneal metastases of colorectal origin with complete cytoreduction followed by HIPEC was reported in phase III prospective-randomized trial [1]. More recently, other groups have improved these results in PM of other origins. Finally, this strategy is now performed at many institutions. Recent studies show that CRS plus intraperitoneal chemotherapy applications confers a prolonged survival in patients with PM of colorectal, gastric, ovarian, and appendiceal neoplasms [1–3] and complete cytoreduction was the most important prognostic factor. In addition to this, volume of peritoneal metastasis (peritoneal cancer index; PCI), biological behavior, histopathological type and grade of tumor, and used chemotherapeutic agents were additional significant prognostic factors in patients with PM.

Besides these improvements, the long-term outcome of these patients is still not satisfied. Further studies need to be conducted with pharmacokinetics of chemotherapeutics and molecular biology studies to develop new therapeutic approaches in this comprehensive strategy.

This special issue of peritoneal metastases offers 18 papers, which consists of 3 papers about pharmacokinetics during intrapertieoneal chemotherapy, 3 about new methods of chemotherapy, 2 about mechanisms and treatment of pulmonary/pleural metastases from peritoneal metastases of appendiceal neoplasm, 3 about new surgical methods and concept, 3 about prevention of recurrence after CC-0 resection, 2 about laparoscopic diagnosis and treatment, and 2 about postoperative morbidity and mortality. These topics covered point to new directions in peritoneal metastases due to gastric, colorectal, appendiceal, and ovarian cancer.

Based on the present results, we have to continue our research and interventions to conquer this refractory disease.

I have to express my deep gratitude to all the guest editors and expert colleagues all over the world who contributed to the special issue.

*Yutaka Yonemura* *Yutaka Yonemura* *Yan Li* *Yan Li* *Paul H. Sugarbaker* *Paul H. Sugarbaker* *Pompiliu Piso* *Pompiliu Piso*

## References

[1] Verwaal VJ, Van Ruth S, De Bree E (2003). Randomized trial of cytoreduction and hyperthermic intraperitoneal chemotherapy versus systemic chemotherapy and palliative surgery in patients with peritoneal carcinomatosis of colorectal cancer. *Journal of Clinical Oncology*.

[2] Yonemura Y, Elnemr A, Endou Y (2012). Effects of neoadjuvant intraperitoneal/systemic chemotherapy (bidirectional chemotherapy) for the treatment of patients with peritoneal metastasis from gastric cancer. *International Journal of Surgical Oncology*.

[3] Sugarbaker PH (2009). Epithelial appendiceal neoplasms. *Cancer Journal*.

